# Meta-virus resource (MetaVR): expanding the frontiers of viral diversity with 24 million uncultivated virus genomes

**DOI:** 10.1093/nar/gkaf1283

**Published:** 2025-11-28

**Authors:** Mateus B Fiamenghi, Antonio Pedro Camargo, Iro N Chasapi, Fotis A Baltoumas, Simon Roux, Artyom A Egorov, Eleni Aplakidou, Eric Olo Ndela, Yumary M Vasquez, I-Min A Chen, Krishna Palaniappan, T B K Reddy, Supratim Mukherjee, Natalia N Ivanova, Frederik Schulz, Tanja Woyke, Emiley A Eloe-Fadrosh, Georgios A Pavlopoulos, Nikos C Kyrpides

**Affiliations:** DOE Joint Genome Institute, Lawrence Berkeley National Laboratory, Berkeley, CA 94720, United States; DOE Joint Genome Institute, Lawrence Berkeley National Laboratory, Berkeley, CA 94720, United States; Department of Biochemistry, Institute of Chemistry, University of São Paulo, São Paulo, SP, 05508-000, Brazil; Institute for Fundamental Biomedical Research, BSRC “Alexander Fleming”, Vari 16672, Greece; Institute for Fundamental Biomedical Research, BSRC “Alexander Fleming”, Vari 16672, Greece; DOE Joint Genome Institute, Lawrence Berkeley National Laboratory, Berkeley, CA 94720, United States; Department of Experimental Medical Science, Lund University, Lund, SE-221 00, Sweden; Institute for Fundamental Biomedical Research, BSRC “Alexander Fleming”, Vari 16672, Greece; DOE Joint Genome Institute, Lawrence Berkeley National Laboratory, Berkeley, CA 94720, United States; DOE Joint Genome Institute, Lawrence Berkeley National Laboratory, Berkeley, CA 94720, United States; DOE Joint Genome Institute, Lawrence Berkeley National Laboratory, Berkeley, CA 94720, United States; DOE Joint Genome Institute, Lawrence Berkeley National Laboratory, Berkeley, CA 94720, United States; DOE Joint Genome Institute, Lawrence Berkeley National Laboratory, Berkeley, CA 94720, United States; DOE Joint Genome Institute, Lawrence Berkeley National Laboratory, Berkeley, CA 94720, United States; DOE Joint Genome Institute, Lawrence Berkeley National Laboratory, Berkeley, CA 94720, United States; DOE Joint Genome Institute, Lawrence Berkeley National Laboratory, Berkeley, CA 94720, United States; DOE Joint Genome Institute, Lawrence Berkeley National Laboratory, Berkeley, CA 94720, United States; DOE Joint Genome Institute, Lawrence Berkeley National Laboratory, Berkeley, CA 94720, United States; Institute for Fundamental Biomedical Research, BSRC “Alexander Fleming”, Vari 16672, Greece; Department of Computational Biology, Mohamed bin Zayed University of Artificial Intelligence (MBZUAI), Abu Dhabi, United Arab Emirates; DOE Joint Genome Institute, Lawrence Berkeley National Laboratory, Berkeley, CA 94720, United States

## Abstract

Viruses are ubiquitous in all environments and impact host metabolism, evolution, and ecology, although our knowledge of their biodiversity is still extremely limited. Viral diversity from genomic and metagenomic datasets has led to an explosion of uncultivated virus genomes (UViGs) and the development of specialized databases to catalog this viral diversity, though many lack comprehensive integration. Here, we introduce meta-virus resource (MetaVR), the successor of the IMG/VR database, designed to overcome previous limitations such as large-scale querying and programmatic access. Drawing on the increase of publicly available genomes and metagenomes, MetaVR significantly expands viral diversity, now comprising 24,435,662 UViGs, a 57.6% increase from its predecessor, organized into over 12 million viral operational taxonomic units. Key enhancements include the integration of curated eukaryotic host information, the integration of protein clusters and predicted structures for comparative studies, and an API for programmatic data access. Furthermore, MetaVR features an updated taxonomic framework based on ICTV release 39, assignment to Baltimore classes, and enhanced host assignment through novel computational tools like iPHoP. These advancements position MetaVR as a unique resource for exploring viral diversity, evolution, and host interactions across diverse environments. MetaVR can be freely accessed at https://www.meta-virome.org/.

## Graphical Abstract

**Graphical Abstract:**
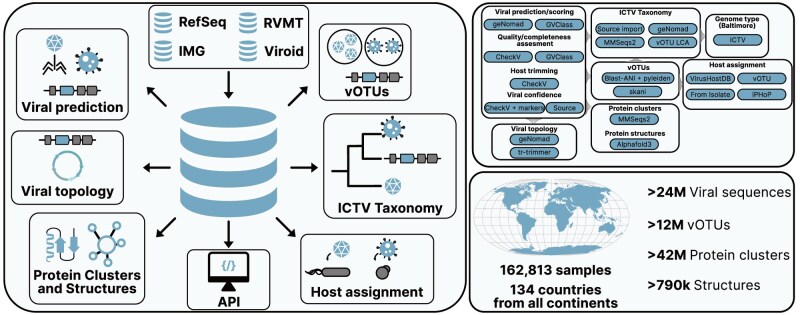

## Introduction

Viruses infect virtually all known life and are largely recognized as important entities that influence the metabolism and ecology of their hosts [1, 2]. This remarkable colonization capability has been driven by diverse evolutionary strategies that have enabled viruses to adopt a wide array of nucleic acid conformations and structural forms [3]. Metagenomics has been established as the primary method for exploring viral diversity [4], and this approach has enabled various discoveries, such as the identification of viral genes that alter host cell processes, insights into virome effects on human and plant health, and enhanced understanding of virus–host interactions [5–9]. Metagenomics has also facilitated the discovery of potential new viruses in hosts of biotechnological, agricultural, and medical interest [10–14]. The cultivation-independent nature of metagenomics streamlines the discovery process and allows for the analysis of far more viral groups than would be possible through cultivation-based approaches alone [6, 7, 9, 15]. The increasing importance of metagenome-derived viral genomes, representing the vast majority of the available uncultivated virus genomes (UViGs), has led to the development of standardized protocols and quality control criteria for their identification, analysis, and sharing in public databases [16].

Advancements in viral discovery due to improved sequencing efforts have led to fragmented viral data resources that impede comparative studies. In response, databases have been developed to collect and curate this diversity, with varying scopes, levels of curation, and amounts of related metadata. Some important resources developed in the last few years include NCBI Viruses [17], a comprehensive public repository of viral sequences from GenBank and RefSeq; ViroidDB [18], a specialized resource for viroids and viroid-like RNAs; RVMT [15], a collection of RNA viruses from metatranscriptomes; PhageScope [19], a resource offering annotation, completeness, and phenotypic data for phages, as well as structure and genome search; and VirjenDB [20], a resource aggregating data from different databases with curated metadata.

Most of these viral databases focus on single viral groups or collection methods, providing comprehensive and detailed information for their specific area. However, this specialization means there is no opportunity for a systematic analysis and comparison of diverse viruses. Consequently, scientists conducting large-scale studies must query multiple sources to gather all necessary data.

To address this gap, the IMG/VR database [21] was launched in 2016 to provide a comprehensive collection across viral groups. Uniquely, it connects to the original IMG/M studies, thereby enriching viral datasets with associated sample metadata and gene annotations from the well-established JGI microbial genome and metagenome annotation pipelines [22].

IMG/VR suffers from some limitations, however, such as difficulties in performing large-scale queries and a lack of robust programmatic access via an API, which have hindered its integration into modern, high-throughput computational workflows.

Here, we present meta-virus resource (MetaVR), the next generation of the IMG/VR database. MetaVR features expanded functionalities and a larger viral collection and aims to represent a comprehensive resource for researchers studying viral diversity, evolution, ecology, and viral impacts on various ecosystems. MetaVR comprises 24 435 662 UViGs, an increase of 57.6% in the number of UViGs compared to IMG/VR v4. We increased the number of external studies with the addition of a large compendium of giant virus MAGs (GVMAG, Vasquez *et al., in prep*) and viral genomes from human gut microbiome samples (UHGV, [23]). Sequences were organized into 12 705 385 viral operational taxonomic units (vOTUs), and 290 450 singleton vOTUs from IMG/VR v4 are now non-singletons. MetaVR also now includes curated eukaryotic host taxonomy for 5188 vOTUs for which this information was available in the VirusHostdb database. To enable large-scale comparative studies and homology search, predicted proteins from all MetaVR UViGs were clustered, and the database now includes these protein clusters together with structural predictions for most clusters with at least 15 unique proteins. MetaVR further extends these capabilities by offering an API for programmatic access and seamless integration into analysis pipelines. Collectively, these advances enable deeper analyses, new discoveries, and an improved understanding of the global virosphere.

## Materials and methods

### Data collection and viral prediction

Uncultivated viral genomes (UViGs) were mined from public assemblies available in IMG/M as of April 2025. In total, 37 961 metagenomic and 8694 metatranscriptomic datasets were processed for viral discovery, as well as 99 377 bacterial and archaeal isolate genomes, 1452 single amplified genomes (SAGs), and 9704 metagenome-assembled genomes (MAGs). For metagenomes, isolate genomes, SAGs, and MAGs, sequences were required to be at least 2 kb long if they contained direct or inverted terminal repeats, and at least 4 kb otherwise. For metatranscriptomes, a uniform 2-kb cutoff was applied. Virus identification was performed using geNomad [24] (version 1.11, parameters: ‘--enable-score-calibration --lenient-taxonomy --full-ictv-lineage --sensitivity 7.0’), employing the score calibration functionality to set the estimated false discovery rate to 2%. Predicted proviruses were processed using CheckV [25] (version 1.0.3) to trim out boundaries and mitigate contamination with host genes. Giant Virus metagenome-assembled genomes (GVMAGs) were predicted through GVClass [26] version 1.0, but their individual scaffolds were also processed through geNomad for the calculation of the viral score through a length-weighted average of all scaffold scores. Sequences from other external studies (RVMT [15], Inoviruses [27], RefSeq [28], and ViroidDB [18]) were also processed through geNomad for viral score assignment.

### UViG topology, completeness and contamination estimation, and provirus quality control

Genome topology was defined by using the assembly source for GVMAGs and sequence predictions for other UViGs. Possible UViG topology values are virus MAG (genomes spanning multiple scaffolds), concatemer (sequences composed of repeats of the same genome unit probably resulting from assembly artifacts), linear (a linear or fragmented contig), provirus (a virus integrated into a host scaffold), direct terminal repeat (DTR), or inverted terminal repeat (ITR) (viral assemblies with repetitive ends, which are putatively complete). Concatemers were predicted using a custom Python script using the average 21-mer frequency (total 21-mers/unique 21-mers) or the number of repeats (using repeat-matcher). If the average 21-mer frequency was >1.4 or if the longest repeat comprised less than 90% of the total sequence length, the genome was considered a concatemer. In a typical sequence, most *k*-mers appear only once, meaning this ratio should be close to 1; thus, a ratio significantly >1 typically reflects [23] an assembly artifact. Similarly, a subsequence considered as a repeat spanning <90% of the total sequence length indicates the presence of multiple short sequence copies, typical of concatemers.

DTRs and ITRs were predicted with tr-trimmer (https://github.com/apcamargo/tr-trimmer, parameters: ‘-l 21 -i -c -a -t -x’). Proviruses were directly obtained from the geNomad predictions and further processed by first trimming regions that encoded ribosomal rRNAs and then using CheckV to remove flanking host genes.

Completeness and contamination of single-contig viruses were estimated using CheckV [25] with default parameters. For GVMAGs, completeness was assessed with GVClass [26]. Unlike CheckV, GVClass does not directly provide contamination estimates as a percentage value but instead uses a duplication factor that is inferred from the copy number of typically low-copy *Nucleocytoviricota* order-level panorthologs. For this reason, the raw GVClass outputs are also made available for further investigation.

### vOTU assignments

Single-contig UViGs newly identified for this database release were subjected to an all-vs-all BLAST [29] analysis (parameters: ‘-task megablast -evalue 1e-5 -max target seqs 20000’). This was followed by the calculation of average nucleotide identity (ANI) and alignment fraction (AF) based on the BLAST results using a custom script (https://bitbucket.org/berkeleylab/checkv/src/master/scripts/anicalc.py). New UViGs to this release were assigned to an IMG/VR v4 vOTU if they exhibited at least 95% ANI and 85% AF to at least 25% of the members of a vOTU. If multiple IMG/VR v4 vOTUs met these criteria, the UViG was allocated to the vOTU with the most members. Remaining UViGs without assignment were subsequently clustered utilizing the Leiden algorithm with default parameters as implemented in the pyLeiden package (https://github.com/apcamargo/pyleiden). This clustering was performed by filtering the BLAST results for pairs satisfying the 95% ANI and 85% AF threshold and using the product of ANI and AF as weights for graph construction.

GVMAGs were clustered separately based on 95% ANI using skani [30], and clusters were directly imported as vOTUs.

### Virus prediction confidence and UViG quality assessment

UViGs predicted with geNomad were considered as being high confidence based on any of the following series of criteria that are orthogonal to the classification process:

- If the UViG was assigned to the same vOTU as a virus obtained from RefSeq;
- If the UViG encoded at least 2 viral marker genes, as determined by geNomad;
- If the UViG encoded at least one viral marker gene and either had a high-confidence/medium-confidence AAI-based completeness estimate by CheckV or a DTR detected.

UViGs obtained from external studies (GVMAGs, RVMT, Inoviruses, viroids, and from RefSeq) were always considered as high confidence. Confidence levels for sequences from the UHGV study were directly imported.

UViG quality was assessed through CheckV v1.0.3, with the exception of those from the UHGV project, from which the quality was directly imported. One important caveat is that CheckV is tailored for single-contig viruses, and thus the quality of GVMAGs should be interpreted with caution.

We evaluated these confidence criteria through two approaches. First, we checked all 5625 RefSeq-imported UViGs against the current and previous IMG/VR v4 confidence parameters and found that 84% and 81% were classified as high-confidence, respectively. Second, we evaluated updated and prior parameters by running geNomad on 154 680 putatively complete plasmid sequences from IMG/PR [31] and 72 556 plasmid sequences from PLSDB [32]. The results show that our confidence criteria do not overpredict viral sequences: only 41 and 40 IMG/PR plasmids were classified as high-confidence viruses using the current and previous parameters, respectively. In contrast, 3632 and 3649 PLSDB plasmids were identified as high-confidence viruses with the current and previous parameters, respectively. It is important to note that these “mispredicted” sequences could also be other types of MGE, such as the recently described phage-plasmids [33], as they carry both viral and plasmid hallmark genes.

### Taxonomic assignment

UViG taxonomic assignments were based on the most specific rank available in ICTV MSL39.v4 and followed these criteria:

- UViGs from GVMAGs, RVMT, viroids, and RefSeq were assigned taxonomy directly from their respective data sources, adjusting the lineage to ICTV’s MSL39 whenever needed.
- UViGs sharing a vOTU with external sources received the lowest common ancestor taxonomic assignment from all UViGs within that vOTU.
- For UViGs without an assignment from the methods above, taxonomy was determined through geNomad.
- Any remaining UViGs without assignment underwent MMseqs2 taxonomy analysis using a custom pipeline and protein database decorated with ICTV taxonomy (available at https://github.com/apcamargo/ictv-taxonomy-challenge-nr).

### Host assignment

Single-contig UViG host assignment was done through multiple approaches:

- If the UViG was identified within the genome assembly of an isolate or SAG, it received the GTDB taxonomy of the source genome.
- For viruses obtained from RefSeq, the host and its taxonomy were retrieved from VirusHostDB release 228 (3 January 2025), including for eukaryotic hosts.
- Viruses still without a host received the consensus taxonomic assignment from their respective vOTU through the find_majority_vote function of the taxopy package (https://github.com/apcamargo/taxopy). The weights parameter of find_majority_vote was used to prioritize eukaryotic hosts over prokaryotic hosts when both groups were present as possible hosts in the vOTU by setting the weight for prokaryotes as 1 and for eukaryotes as 2. This was done due to the eukaryotic prediction originating from curated data from VirusHostDB and to avoid possible cases of contamination or mislabeling from isolate sequencing.
- Any remaining UViGs without assignment were subjected to iPHoP [34] v1.4.1 with the iPHoP_db_Jun25_rw database for *de novo* host prediction. For predictions with a score of at least 90, the UViG’s host was predicted as the corresponding genus, while for predictions with a score <90 but at least 75, the UViG’s host was predicted as the corresponding family.

### Clustering of predicted proteins

Protein sequences were clustered with MMseqs2 (version 14.7e284) [35] using a two-stage procedure. First, we performed an all-vs-all comparison requiring ≥85% bidirectional coverage, then grouped sequences with the greedy set-cover method (parameters: ‘cluster -s 5.6 -c 0.85 --cluster-steps 3 --kmer-per-seq 50’). For each preliminary cluster, MMseqs2’s center-star routine was used to produce a multiple sequence alignment that was converted to a protein profile from which consensus sequences were derived. These sequences were then queried against the protein set in a sequence-vs-profile search with ≥ 90% bidirectional coverage (parameters: ‘search -s 6.6 -c 0.9 -e 1e-5 --add-self-matches’), which guided the second clustering step. Final multiple sequence alignments for each cluster were generated with FAMSA v2.2.3 [36].

### Protein structure prediction

Structures were predicted for representative sequences of protein clusters with at least 15 unique members due to computational constraints. First, the multiple sequence alignment (MSA) of each cluster was enriched using MMseqs2 v. 17-b804f  [35] by searching for similar sequences against the following databases: PDB  [37], UniRef90  [38, 39], and the MetaVR cluster MSAs (parameters: ‘-e 1e-5 --max-seqs 100000 -s 7 --num-iterations 2’). Results of the search were then converted to MSAs in a3m format with reduced redundancy using MMseqs2’s result2msa module (parameters: ‘--max-seq-id 0.9’). Finally, AlphaFold (version 3.0.1) [40] was used to predict monomer structures of the representative proteins, with precomputed MSAs provided as input (parameters: ‘--norun_data_pipeline’). The top-ranking prediction for each cluster was taken for subsequent analyses.

Foldseek [41] (version 10-941cd33) was used to cluster MetaVR’s structures in TM-align mode (parameters: ‘--alignment-type 1 -c 0.8 --tmscore-threshold 0.4 -e 0.001’), both individually and in conjunction with the Viral AlphaFold Database (VAD) [42] and Big Fantastic Virus Database (BFVD) [43].

### Database Implementation

MetaVR is implemented using the ASP.NET Core Model–View–Controller (MVC) framework, with MariaDB as its database management system. The back-end is coded in C#, while the front-end employs HTML, CSS, and JavaScript. The system is divided into Models, Views, Controllers, Services, and Factories. Models, represented by C# classes, define data and apply validation rules. Views use Razor (.cshtml) templates and C# logic to format data and generate HTML. The framework allows for asynchronous parallel execution of tasks, enabling multiple concurrent queries to the underlying database. Additional website functionality is provided by integrating a suite of plugins, viewers, and analysis tools. Genome sequence search is performed using NCBI BLAST+ [29], while protein queries are performed using DIAMOND [44] for sequences and Reseek [45] for 3D structure models. These search jobs are handled by a workload manager, where each query is placed in a job queue and receives a unique job identifier, which users can then bookmark to return later and view their results. The Mol* viewer [46] is used to render 3D models of predicted structures in the protein cluster entry pages. Finally, the OpenStreetMap API is used to support geospatial data representation. The MetaVR API is implemented in Python, using FastAPI for the backend and Swagger to render the interactive frontend. Furthermore, limitations are imposed on the API routes to ensure seamless usage for all users. API routes can be accessed interactively through https://meta-virome.org/api/docs or programmatically as GET and POST requests.

## Results

### Novel features in MetaVR

MetaVR presents several novel features compared to the IMG/VR v4 release, namely:

- An overall increase in the number of UViG sequences, including a 57.6% increase of total UViGs, a 48% increase in total vOTUs, a 7.6-fold increase in GVMAG UViGs, and a 3.8-fold increase in GVMAG vOTUs;
- An updated taxonomy, based on ICTV release 39;
- The addition of a Baltimore classification assignment (virus genome type) for viruses with taxonomic information;
- Updated host connection, using iPHoP for computational prediction;
- The addition of protein clusters and protein structure predictions;
- A new MetaVR portal with an updated UI and an API allowing programmatic access of the data.

We detail these improvements in the sections below.

### MetaVR greatly increases the known viral diversity

**Figure 1.**
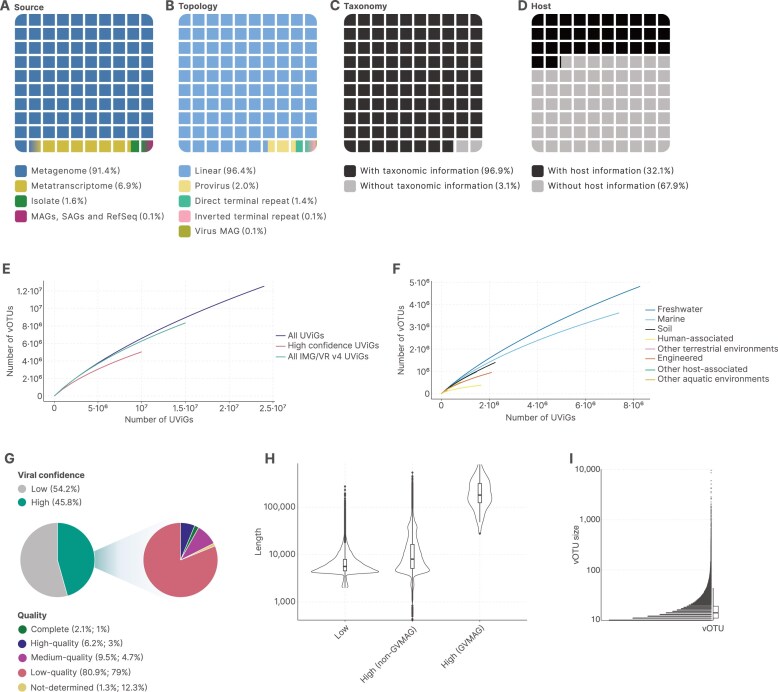
**(A-D)** Distribution of UViGs in the MetaVR database based on their source (**A**), predicted topology (**B**), assigned taxonomy (**C**), and predicted or assigned host (**D**). **(E, F)** Species accumulation curves comparing high-confidence UViGs and all UViGs from the IMG/VR v4 and MetaVR releases (**E**), and per assigned environment **(F)**, based on the GOLD classification. (**G**) Distribution of UViGs in accordance with their assigned viral confidence. High-confidence viruses were also stratified in accordance with CheckV quality tiers. Left percentages represent the fraction of high-confidence UViGs at that quality tier, while right percentages represent the fraction of UViGs when considering the whole database. (**H**) Length distributions of UViGs with low and high viral confidence predictions. (**I**) Size distribution for vOTUs with at least 10 members

MetaVR expands on the number of sequences from IMG/VR v4 by analyzing novel metagenomes and metatranscriptomes added to IMG since its previous release using an updated version of geNomad, which includes several more marker genes to facilitate the identification of newer viral groups. We also incorporate new viral sequences from RefSeq release 228 and Virus MAGs, identified using an updated version of GVClass (see the “Materials and methods” section). In total, MetaVR now includes 5625 genomes from RefSeq and 24 435 662 UViGs (a 57% increase over the IMG/VR v4 release) identified across 37 961 metagenomes, 8694 metatranscriptomes, and 99 377 genomes of isolated prokaryotes, 1452 SAGs, and 9704 MAGs (Fig. 1A). The majority of these UViGs are linear sequences (Fig. 1B) and could be assigned to a taxonomic rank (Fig. 1C). Moreover, 7 833 811 UViGs have a host assignment (Fig. 1D).

By leveraging study metadata from the IMG/M database, we could directly connect viral communities to the environments they are found in, observing that most viral sequences originated from samples collected in marine and freshwater environments, both of which are known to be large viral hotspots [47–49]. UViGs were clustered at 95% ANI and 85% AF into 12 705 385 vOTUs (∼69% singletons), which represent a 1.45-fold increase over IMG/VR v4. Although this expanded database represents a 56% increase in viral sequences from the last database release, we are still far from reaching saturation both overall and when considering individual environments (Fig. 1E and F). As seen in previous database releases [50, 51], it is estimated that a large number of virus genomes are yet to be discovered.

Due to the inherent uncertainty that computational predictions may bring to viral discovery pipelines [52], it is important to distinguish sequences that are highly probable to be viral (due to having multiple genes with functions that are known to be associated with viruses) and sequences that are uncertain (a sequence without sufficient genetic information for conclusive evidence but still flagged as viral due to machine learning or other methods based on sequence signatures). Compared to previous versions of the IMG/VR database, we have transformed the “high confidence” UViG attribute from IMG/VR v4 into a “viral confidence” field, which indicates whether the virus identification for a given UViG is supported by orthogonal information, discriminating high-confidence and low-confidence viral genome identification. This release comprises 13 243 051 low-confidence sequences and 11 192 611 high-confidence sequences. Notably, 1 774 978 sequences from the IMG/VR v4 release that were considered low-confidence are now marked as high-confidence due to simplified discrimination parameters compared to IMG/VR v4 (e.g. CRISPR spacers are not used for this release, and medium-quality CheckV results are considered more highly for all parameters; see the “Materials and methods” section) coupled with a newer geNomad release that includes novel viral marker genes. We simplified the criteria that we use to label UViGs as high-confidence to make this flag more interpretable. Nevertheless, to allow users to manually curate sequences of interest, we have included the rationale for each sequence to be included on the high-confidence dataset (Supplementary Tables S1 and S2).

Low-confidence sequences come with a higher chance of being mispredictions, yet can drive the discovery of novel viral groups or genes due to their limited similarity to known viral elements, and they may represent evolutionary divergence or unique genetic adaptations still uncaptured in viral databases [53]. Regarding the high-confidence portion, 232 103 sequences were classified as complete, 695 060 as high quality, 1 062 269 as medium quality, 9 058 553 as low quality, and 144 626 as not determined, according to the MIUViG thresholds and CheckV completeness estimates (Fig. 1G).

**Figure 2.**
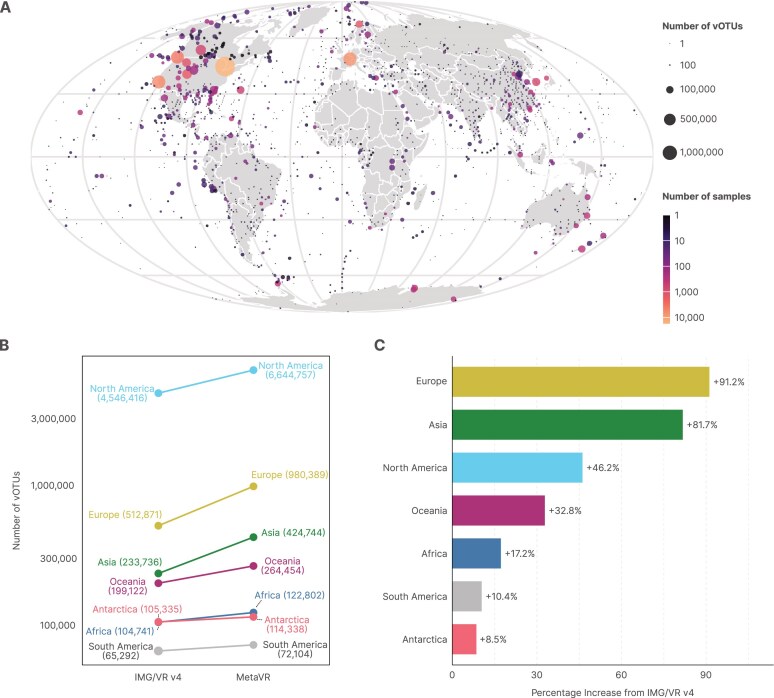
(**A**) Distribution of viruses in MetaVR at the vOTU level based on the coordinates of their respective samples submitted to IMG/M and shown using a Mollweide projection. Larger circles on the map indicate locations with a higher number of vOTUs, and these circles are color-coded according to the number of samples (datasets). For enhanced visualization, samples located within 5100 km of each other were grouped by their latitude and longitude, and their medoid coordinates were used for plotting. (**B**) Increase in the absolute number of vOTUs for each continent between IMG/VR v4 and MetaVR. (**C**) Percentage increase in the number of vOTUs per continent between IMG/VR v4 and MetaVR

Sequences from MetaVR were sampled from all around the world, with a bias toward North America and Europe (Fig. 2A). Overall, we noticed an increase in vOTUs from all continents when compared to the IMG/VR v4 release (Fig. 2B), with the largest increase in vOTUs coming from Europe and Asia (Fig. 2C). Nevertheless, there remains a need for additional sequencing from underrepresented regions like South America and Africa.

### Enabling the study of viral evolution through an updated taxonomy and host information framework

**Figure 3.**
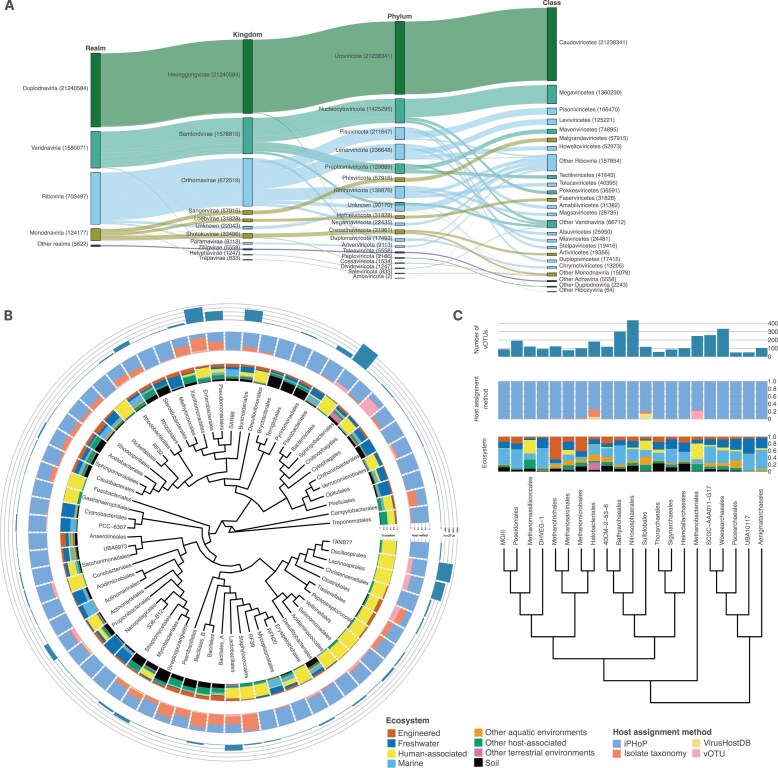
(**A**) Alluvial plot illustrating the main taxonomic realms, kingdoms, phyla, and classes represented in MetaVR. The top 20 classes are individually displayed, with the remaining classes grouped as “Others.” (B, C) Cladogram depicting the GTDB r226, pruned at the order level for bacteria (**B**) and archaea (**C**). Only host classes with at least 200 vOTUs for bacteria and 50 vOTUs for archaea are included, filtered to show only high-quality and complete UViGs. Both cladograms are augmented with the number of vOTUs associated with each specific class as its host (outermost graph), the proportion of each host assignment method used to link that host to a virus (middle graph), and the proportion of each environment in which the UViG was discovered (innermost graph).

Viral taxonomy for MetaVR is based on ICTV Taxonomy Release 39. Notably, 23 668 185 UViGs (∼97%) from 12 271 957 vOTUs (∼97%) were assigned a taxonomic label for at least one rank. When considering only high-confidence viruses, only 0.4% of sequences are lacking a taxonomic classification, and 1 843 479 UViGs from IMG/VR v4 now have a more specific rank in MetaVR, 220 949 (170 774 vOTUs) of which had no prior taxonomic assignment. Most UViGs are assigned to the *Caudoviricetes* class of tailed dsDNA bacteriophages and archaeal viruses (96%; Fig. 3A). The GVMAG catalog (all assigned to the *Nucleocytoviricota* phylum) has increased by almost eight times in the number of UViGs, growing from 2055 to 15710 sequences, and from 2005 to 8900 vOTUs. Additionally, MetaVR benefits from an update to our virus discovery tool, geNomad, resulting in an expanded collection of different groups that were often missed before, such as *Finnlakeviridae* (*Varidnaviria*), *Pleolipoviridae* (*Monodnaviria*), and some inovirus clades (*Faserviricetes*). Exemplifying this, we identified 229 sequences (129 vOTUs) assigned by geNomad to the FliP virus group (*Finnlakeviridae*) for which only two genomes are currently available in NCBI GenBank [54], including 55 considered as high-confidence predictions and 42 putatively complete.

The improved taxonomic assignment has facilitated the inclusion of genome-type-based (Baltimore) classification [3] to UViGs, which will enable researchers to screen sequences of interest based on their nucleic acid content and type and could help in identifying the emergence of viral lineages and their evolution [55].

The MetaVR database also includes improved host prediction over previous IMG/VR releases. Of note is the inclusion of eukaryotic host taxonomy for UViGs by connecting the information obtained from VirusHostDB (RefSeq viruses), which we believe will allow researchers to explore viral diversity across a broader range of biological systems. By assigning hosts through vOTU connection, we were able to connect eukaryote hosts to an additional 2292 UViGs. Furthermore, we now use iPHoP [34] to predict prokaryote hosts. iPHoP is a viral host prediction tool that aggregates and harmonizes the results of different methods for host prediction and predicts the most probable host for the viral sequence. This novel strategy has enabled the assignment of putative hosts to 7 833 811 UViGs (4 321 235 vOTUs), including 49 812 UViGs (40 199 vOTUs) from the IMG/VR v4 release that previously lacked host information. Prokaryote hosts for all UViGs have been updated to the GTDB r226 taxonomy (Fig. 3B and C).

### Enabling large-scale functional studies with protein clusters and structures

**Figure 4.**
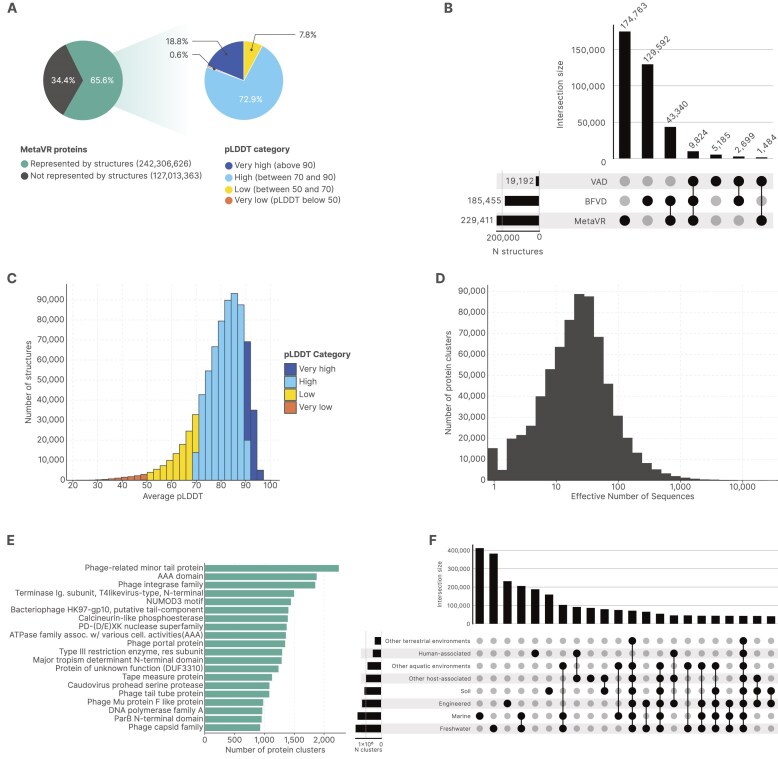
**A**) Pie charts demonstrating the proportion of proteins of the MetaVR database represented by structural predictions and the proportion in each pLDDT (predicted local distance difference test) category. (**B**) Upset plot showing the limited overlap of structural clusters across the MetaVR, BFVD, and VAD databases. Clustering was performed using FoldSeek in TM-alignment mode. (**C**) Histogram showing the average pLDDT score for each predicted protein structure. (**D**) Histogram depicting the effective number of sequences within each protein cluster, as computed by NEFFy [58]. (**E**) Most prevalent Pfam geNomad viral hallmark annotations for protein clusters with a predicted structure. Annotations were inferred with Pfam [59] release 37.4 with gathering thresholds; the best hit for each protein was kept, and the most prevalent annotation was transferred to the cluster. Only clusters with Pfam annotations are shown. (**F**) Upset plot illustrating the number of protein clusters exclusively found in or shared between different environments. Only clusters containing at least 10 proteins and with a minimum intersection size of 35 000 are shown.

The most significant additions to MetaVR compared with previous database releases are protein clusters and structures. Protein clusters, built *de novo* based on all predicted protein sequences across MetaVR genomes, facilitate large-scale functional inference, as it allows the propagation of annotations from known members to entire families and makes computationally intensive analyses feasible across the dataset. Structures allow for improved annotation of functions of proteins, as comparing the structure of a novel protein to known structures can reveal evolutionary relationships and conserved functional elements, even in the absence of significant sequence similarity [56]. In total, we obtained 42 390 306 unique protein clusters, from which 748 927 structures (79.7% mean pLDDT; 81.6% median pLDDT) were predicted with AlphaFold3 [40], which makes MetaVR the largest repository of predicted virus protein structures to date [43, 57]. Over 65% of the total protein set for MetaVR is represented by these structures (Fig. 4A), and over 70% of the geNomad marker genes with Pfam annotations are represented by structures. When compared to other large databases of predicted protein structures (BFVD and VAD), the structures obtained seem to be mostly distinct and not closely related between databases, emphasizing the novel protein diversity being sampled and made available in MetaVR (Fig. 4B). While most protein clusters contain few sequences or are singletons, a substantial number are large and sufficiently diverse to lead to high-quality structure prediction, as reflected in their high effective number of sequences [58] and the high average pLDDT values of the resulting structures (Fig. 4C and D). Overall, 11 416 distinct Pfam domains were detected in the protein clusters with structural prediction, including functions related to various aspects of viral replication and infection cycles. The most prevalent annotations for geNomad viral hallmarks can be seen in Fig. 4E. Many clusters are environment-specific, particularly those found in aquatic environments and human-associated microbiomes (Fig. 4F).

### MetaVR as a new interface to search and access large viral genome catalogs

**Figure 5.**
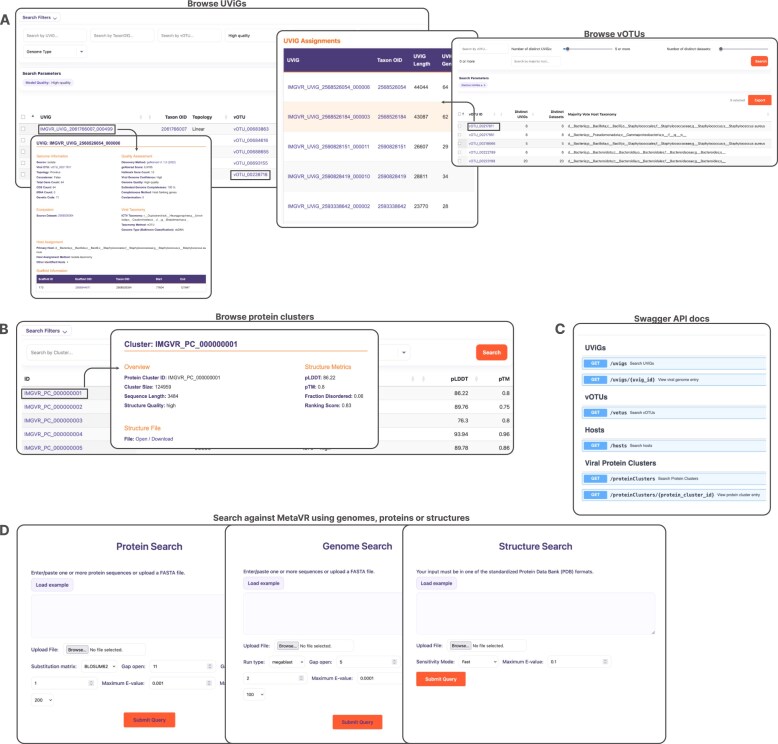
(**A**) The MetaVR user interface facilitates browsing by UViGs and vOTUs, offering direct data export for custom pipeline integration. Users can add filters directly from this page, minimizing navigation for query adjustments. Clicking on any vOTU or UViG provides access to a dedicated page for further investigation. (**B**) A similar interface is implemented for protein clusters, where clicking on a cluster opens a specific page for visualizing and downloading its predicted structure and allows exploration of viral proteins within that cluster. (**C**) MetaVR offers API access compliant with the OpenAPI schema. (**D**) Users can search MetaVR using their own viral proteins, genomes, or predicted structures.

The MetaVR database is made available through a new web interface (https://www.meta-virome.org/) that enables large-scale search of UViGs, vOTUs, protein clusters, and predicted structures. Researchers can navigate and query the collections of protein sequences, as well as examine sequence similarities, identify conserved domains, explore phylogenetic relationships, and understand potential functional annotations (Fig. 5). Additionally, protein cluster pages allow the visualization and export of the predicted structures. Users can also upload sequences to query against genomes using BLAST [29], the protein clusters using DIAMOND [44], and structures using Reseek [45]. Finally, the MetaVR database is now available through API endpoints (https://meta-virome.org/api/docs), allowing users to programmatically access and filter data and integrate into their own analysis pipelines.

## Conclusion

Genomes from UViGs have reshaped the field of viromics, enabling large-scale viral discovery and analysis independent of cultivation efforts. Although these genomes quickly became the primary framework to study the vast viral diversity, there are still caveats to their use that warrant care from researchers. While the prediction accuracy has increased over the past years, there are uncertainties associated with any computational prediction tool, and thus some caution must be exercised in regard to possible false positives. We thus strongly encourage MetaVR users to leverage the different uncertainty thresholds provided to classify UViGs into high- and low-confidence sequences, as well as the UViG quality estimation, and use the most appropriate MetaVR subset for their analysis. Efforts related to community curation, sequencing samples from undersampled regions, or experimentally validating selected predictions can mitigate biases and false discoveries.

Furthermore, as discussed previously [50], computational host assignment for viral sequences remains a challenge. Since the last database release, there have been significant advances, and the addition of predictions from iPHoP in MetaVR helped assign putative hosts to 7 049 110 UViGs (4 173 478 vOTUs). Caution must be taken to interpret these results, as these remain *in silico* host predictions with estimated false discovery rates of ∼5%–10%; however, we believe that this extended framework for host assignment will drive deeper biological insights into host-virus relationships and how these interactions shape ecosystem dynamics in the environments they are found in.

The most significant expansion in MetaVR compared to its predecessor IMG/VR v4 is the addition of protein clusters and structures for representative sequences. This framework will allow studies in evolutionary origins of proteins, help improve functional and domain annotation, and protein-protein interactions. Notably, it has been shown that comparing structures can allow for the annotation of more distantly related sequences with greater accuracy [56].

Despite the increased amount of sequences in MetaVR, the still unsaturated biodiversity estimates and the presence of low-confidence sequences underscore the ongoing need for improved computational prediction methods and further exploration of underrepresented environments. With this in mind, we believe that MetaVR will continue to serve as a key resource for researchers investigating viral evolution, ecology, and host interactions, facilitating deeper insights into the global virome.

## Supplementary Material

gkaf1283_Supplemental_Files

## Data Availability

MetaVR is publicly available at https://www.meta-virome.org/. Code related to analysis and database generation can be found at https://code.jgi.doe.gov/antoniop.camargo/metavr/.
